# Accuracy of Optic Nerve Sheath Diameter Measurements in Pocket-Sized Ultrasound Devices in a Simulation Model

**DOI:** 10.3389/fmed.2022.831778

**Published:** 2022-03-02

**Authors:** Garrett G. R. J. Johnson, Tomislav Jelic, Angela Derksen, Bertram Unger, Frederick A. Zeiler, Markus T. Ziesmann, Lawrence M. Gillman

**Affiliations:** ^1^Department of Surgery, University of Manitoba, Winnipeg, MB, Canada; ^2^Department of Emergency Medicine, University of Manitoba, Winnipeg, MB, Canada; ^3^Emergency Department, Seven Oaks General Hospital, Winnipeg, MB, Canada; ^4^Department of Internal Medicine, University of Manitoba, Winnipeg, MB, Canada; ^5^Section of Neurosurgery, Department of Surgery, University of Manitoba, Winnipeg, MB, Canada; ^6^Department of Anatomy and Cell Science, University of Manitoba, Winnipeg, MB, Canada; ^7^Biomedical Engineering, Faculty of Engineering, University of Manitoba, Winnipeg, MB, Canada; ^8^Centre on Aging, University of Manitoba, Winnipeg, MB, Canada; ^9^Division of Anaesthesia, Department of Medicine, University of Cambridge, Cambridge, United Kingdom

**Keywords:** ultrasound, intracranial pressure, point-of-care, simulation, intracranial hypertension, optic nerve

## Abstract

**Introduction:**

Transorbital sonographic measurement of optic nerve sheath diameter (ONSD) is an emerging non-invasive technique for the identification and monitoring of intracranial hypertension. In recent years, new pocket ultrasound devices have become available, and it is uncertain if they have the resolution to measure such small structures appropriately as compared to their predecessors. In this study, we measure the performance of three ultrasound units on a simulation model to establish their precision and accuracy.

**Methods:**

ONSD was measured by three expert point-of-care sonographers using ultrasound machines three times on each of seven discrete ONS model sizes ranging from 3.5 to 7.9 mm. Two pocket ultrasounds (IVIZ, Sonosite, and Lumify, Philips) and one standard-sized portable ultrasound (M-Turbo, Sonosite) were used. Measurements were analyzed for mean error and variance and tested for significance using blocked covariance matrix regression analyses.

**Results:**

The devices differed in their variances (Lumify: 0.19 mm^2^, M-Turbo: 0.26 mm^2^, IVIZ: 0.34 mm^2^) and their mean error (Lumify: −0.05 mm, M-Turbo: 0.10 mm, IVIZ: −0.10 mm). The difference in mean error between users is not significant (*p* = 0.45), but there is a significant difference in mean error between devices (*p* = 0.02).

**Conclusions:**

Accurate ONSD measurement is possible utilizing pocket-sized ultrasound, and in some cases, may be more accurate than larger portable ultrasound units. While the differences in these devices were statistically significant, all three were highly accurate, with one pocket device (Lumify) outperforming the rest. Further study in human subjects should be conducted prior to using pocket ultrasound devices for *in vivo* diagnosis of intracranial hypertension.

## Introduction

Intracranial hypertension (IH) is a lethal and relatively common phenomenon in critically ill traumatic brain-injured patients. While these patients' prognosis is guarded, they fair much worse if this condition is not diagnosed accurately and treated promptly (1–3). Current modalities used to diagnose IH include clinical examination, imaging, and invasive intracranial monitors (4). While CT scan and MRI aid in the diagnosis of IH, they are time-consuming and are difficult to administer repeatedly to provide continuous intracranial pressure (ICP) monitoring often required by these patients (5, 6). Physical examination findings alone are not sufficiently sensitive or specific and may be difficult to appreciate in patients with multiple injuries (7, 8). ICP monitoring devices are considered gold standard for the diagnosis and monitoring of IH. Their limitations are that they are invasive, require neurosurgical expertise both to administer and monitor, and are associated with risks including bleeding and infection (9).

Transorbital ultrasound assessment of optic nerve sheath diameter (ONSD) as a non-invasive means to diagnose and monitor patients suspected of IH has been investigated with promising results (10–14). However, point-of-care ultrasound (POCUS) measurements, in general, are operator-dependent (15–20), and some authors question their diagnostic utility (21). A recent metaanalysis has demonstrated high diagnostic accuracy of ONSD across 50 studies (22). The use of POCUS in emergency departments, medical wards, and intensive care units as an adjunct to the physical exam is widespread. This is likely due to improvements in ultrasound technology leading to smaller ultraportable devices, less expensive technology, and the dissemination of ultrasound expertise as the technique becomes more established. However, most literature validating ONSD measurements of ICP has used larger portable ultrasound machines (15). It is unclear how this modality can translate to newer handheld machines, which may have a limited range of probe frequencies, lower penetration, touch screens, smaller screen sizes, and varying screen resolutions. ONSD measurement by ultrasound requires accurate measurement of a subcentimeter structure to the nearest 0.1 mm (19), and it is unclear if operators using these newer ultrasound devices can perform this task effectively. In the past, we have shown, using a simulation model, that one pocket-sized ultrasound device (Vscan, GE Healthcare) performed equal to a standard-sized validated ultrasound (M-turbo, Sonosite) with respect to intra and interobserver variability, and there was a high level of agreement between measurements obtained using the two machines (15). However, each new pocket ultrasound device is unique, with differing manufacturer settings, screen sizes, and probe frequencies, and therefore should be tested as they become available prior to using it for this critical diagnostic purpose. The present study aimed to compare two newer hand-held ultrasound units (Sonosite IVIZ and Philips Lumify) to a previously validated portable unit (Sonosite M-Turbo) utilizing a simulation model of the ONS and to determine their relative measurement errors and variance.

## Materials and Methods

### Ethics

The study protocol was submitted to the University of Manitoba Health Research Ethics Board and determined not to require ethics review.

### Study Design

**Figure 1:**
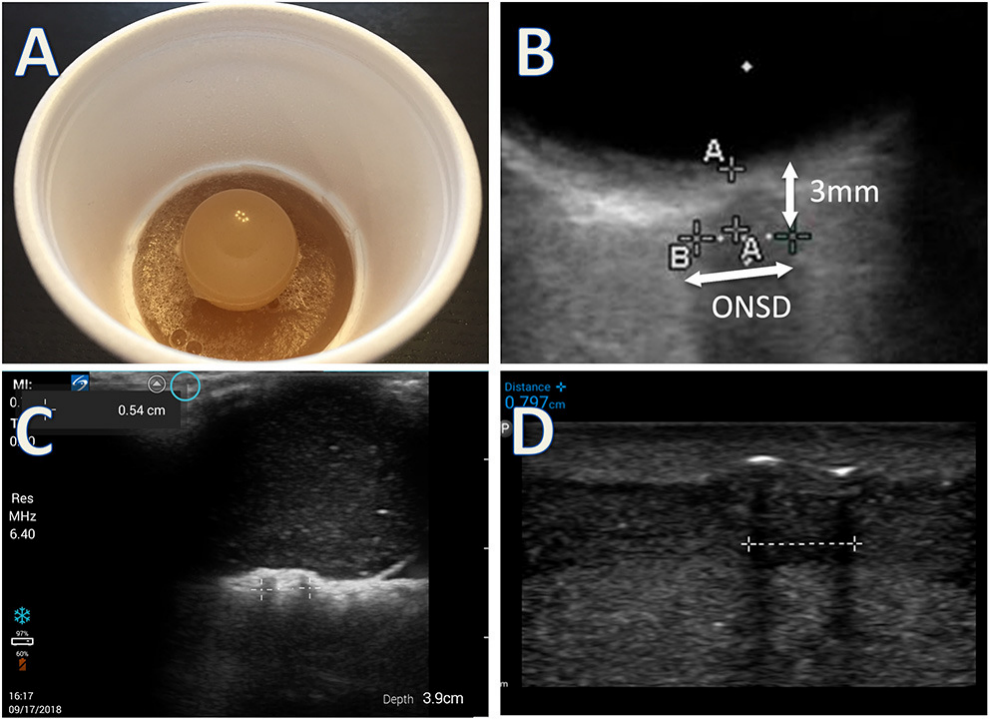
**(A)** Simulation model made with a short segment of intravenous or nasogastric tube suspended in gelatin, under a gelatin eyeball. **(B)** Image of simulation model obtained from Onsite M-turbo (standard sized portable ultrasound) zoomed in with superimposed arrows to highlight measurement technique. **(C)** Image of simulation model from Sonosite IVIZ (pocket ultrasound) device. **(D)** Image of simulation model from Philips Lumify (pocket ultrasound) device. All measurements performed at 3 mm behind the simulated orbit. Calipers used to measure distance behind the globe not shown in **(C,D)**.

Measurements were made using a simulation model of the ONS as published previously (23) (Figure 1). In brief, medical tubing of various diameters was cut to 1 cm in length, encased in an opaque gelatin-psyllium powder matrix, and placed in Styrofoam^TM^ coffee cups. The common B-scan ultrasound technique for measuring ONSD in the literature was used (11, 12, 18, 24, 25). This is performed by placement of the probe over the simulated orbit to identify an axial image of the ONS behind the globe. The digital calipers are used to identify a position 3 mm behind the optic disk, at which point the ONSD is measured. Three ultrasound units were compared: Philips Lumify^TM^ with linear 12–4 MHz probe using the vascular preset and a depth of 2–4 cm, (Koninklijke Philips N.V., Amsterdam, Netherlands) connected to a Samsung Galaxy Tab S2^TM^ (Samsung Group, Seoul, South Korea); Sonosite IVIZ^TM^ with linear 10–5 MHz probe using the vascular preset and a depth of 2–4 cm(SonoSite Inc, Bothell, WA, USA); and Sonosite M-Turbo^TM^ with 13–6 MHz linear array using the ophthalmic preset and a depth of 2–4 cm(SonoSite Inc, Bothell, WA, USA). The ophthalmic presets did not exist for the two pocket devices. The vascular presets were selected for these models because they most closely approximated the image quality of the ophthalmic preset. For all three devices, the user cannot directly control the ultrasound output power. Rather the system does this automatically to limit mechanical index (MI) and thermal index (TI) below the maximum thresholds for the selected presets. Power outputs were monitored intermittently for all three ultrasound devices to assess whether they could stay below FDA safety thresholds for ophthalmic use. Gain settings were adjusted by the operators in real time to attain the clearest images. Sonographers obtained their own images and measured each model in random order with each machine three times. Randomization was performed by assigning each model in a computer-generated random sequence, about which participants were unaware. All models appeared identical (Figure 1A), identifiable only by a letter written on the bottom of the coffee cup. Operators were blinded to the model's identity, the actual diameters of the tubing, and to each other's measurements. Trials were blocked by machine so that each operator made all measurements with one machine before moving on to the next. The order of the machine each operator used in sequence was also random and was predetermined by computer-generated random sequence.

### Participants

Measurements were conducted by three POCUS experts (LG, TJ, and AD) in a single 3 h session. Experts were physicians with additional POCUS training including ONSD measurement training (fellowship or ultrasound courses), and who use POCUS in their usual clinical practice. However, none of these experts perform ONSD measurements in their typical clinical practice as this technique is largely considered to be investigational in our center.

### Sample Size

We defined a difference in the measurement error of 0.5 mm or less between devices as equivalent. Using the B-scan technique, many authors have examined the range in “normal” ONSD values in healthy subjects. The standard deviation of the mean normal ONSD size varies by ≥0.5 mm in most of these studies (20, 26–28). Therefore, as a SD ± 0.5 mm is inherent to the technique, due to variations in normal anatomy, this threshold was chosen as the cutoff for acceptable variation between devices. We felt that a smaller difference between devices was unlikely to be clinically significant. The expected standard deviation was set at 0.51 mm, based on a prior study using the same simulation models and experts (23). Significance was set at 0.05, and power at 90%, to calculate a required sample size of at least 23 measurements per ultrasound device (29). Seven models were made using various tube diameters to simulate the range of ONSD sizes found *in vivo* (13, 19). The diameters were 3.5 mm (polyvinyl intravenous line tubing), 4 mm (nasogastric tube), 4.2 mm (spring wire guide sheath), 4.6 mm (nasogastric tube), 5.5 mm (nasogastric tube), 6 mm (tracheostomy canula), and 7.9 mm (nasogastric tube). Sizes of tubes were confirmed using digital calipers (Neiko 01407A, Neiko®, Taiwan, ROC) calibrated to the nearest 0.1 mm. Tube diameters were verified immediately prior to data collection, and again at the end of the study (and remained identical). The diameters were chosen so they clustered around 4–6 mm as this is where an ultrasonographer would have to discriminate between normal and abnormal ONSD (18). There were seven models, three sonographers, and three ultrasound machines. Each measurement was performed in triplicate. There were a total of 189 measurements performed, with 63 measurements per ultrasound device.

### Outcomes

Study primary outcomes were measurement error and variance stratified by the ultrasound machine. Ultrasound user measurement error and error stratified by model were measured to account for heterogeneity between operators or models.

### Data Analysis

All statistical analyses were performed using SAS version 9.3 (SAS Institute, Cary NC, USA). Figures were generated using SPSS Statistics version 27 (IBM, Armonk, NY, USA). Ultrasound measurements were compared to tube diameter as measured separately with digital calipers. Previous determinations of ONSD measurement accuracy have relied upon Bland–Altman agreement analysis to estimate a true ONS size based upon mean measurements (15). By using a simulation model with a physical tube of known diameter, the “true” ONSD can be measured precisely, and thus measurement error can be calculated. Devices were compared for mean squared error, variance, and error (bias). Blocked covariance matrix regression analyses using a series of F-tests based upon maximum likelihood were performed to analyze the effects of device, user, and model size on measurement accuracy.

## Results

### Device Accuracy

The mean squared error for the Philips Lumify, Sonosite M-Turbo, and Sonosite IVIZ were 0.19, 0.27, and 0.35 mm^2^ respectively. The variances were 0.19, 0.26, and 0.34mm^2^ for the Philips, the M-turbo, and the IVIZ respectively. Measurement error stratified by ultrasound machine is shown in Figure 1 and was statistically significant at *p* = 0.02.

### Interobserver and Between Model Variability

Upon regression analysis, the differences in measurement error between users were not significant (*p* = 0.45). Measurement error stratified by the user, and by ONSD size are shown in **Figures 3**, **4** respectively.

### Safety Data

The Philips device had an MI range from 0.4 to 0.9 and a TI from 0.0 to 0.1 during the study period. For the IVIZ, the MI ranged from 0.7–0.8 and the TI from 0.02–0.1. For the M-turbo, MI was 0.2 and TI 0–0.1.

## Discussion

It was previously unknown if new pocket ultrasound devices can be used to make clinically useful ONSD measurements similar to their larger predecessors. Preliminary Vscan data (GE healthcare) were promising (15), but until now other new ultrasound machines have not been tested for this purpose. We aimed to measure the accuracy of ONSD measurements of two newer pocket-sized handheld ultrasound devices (IVIZ and Lumify) and of a conventional sized portable unit (M-turbo). This was achieved by using our ultrasound model, which allowed us to compare actual sheath diameter to the measurements obtained by the ultrasound devices and determine their difference (error) and variance. This study demonstrated that mean squared error is the lowest in the Philips device, followed by the M-turbo, and then the IVIZ, driven largely by the device variance. Interobserver variation was low and not significantly different between operators in this study.

**Figure 2:**
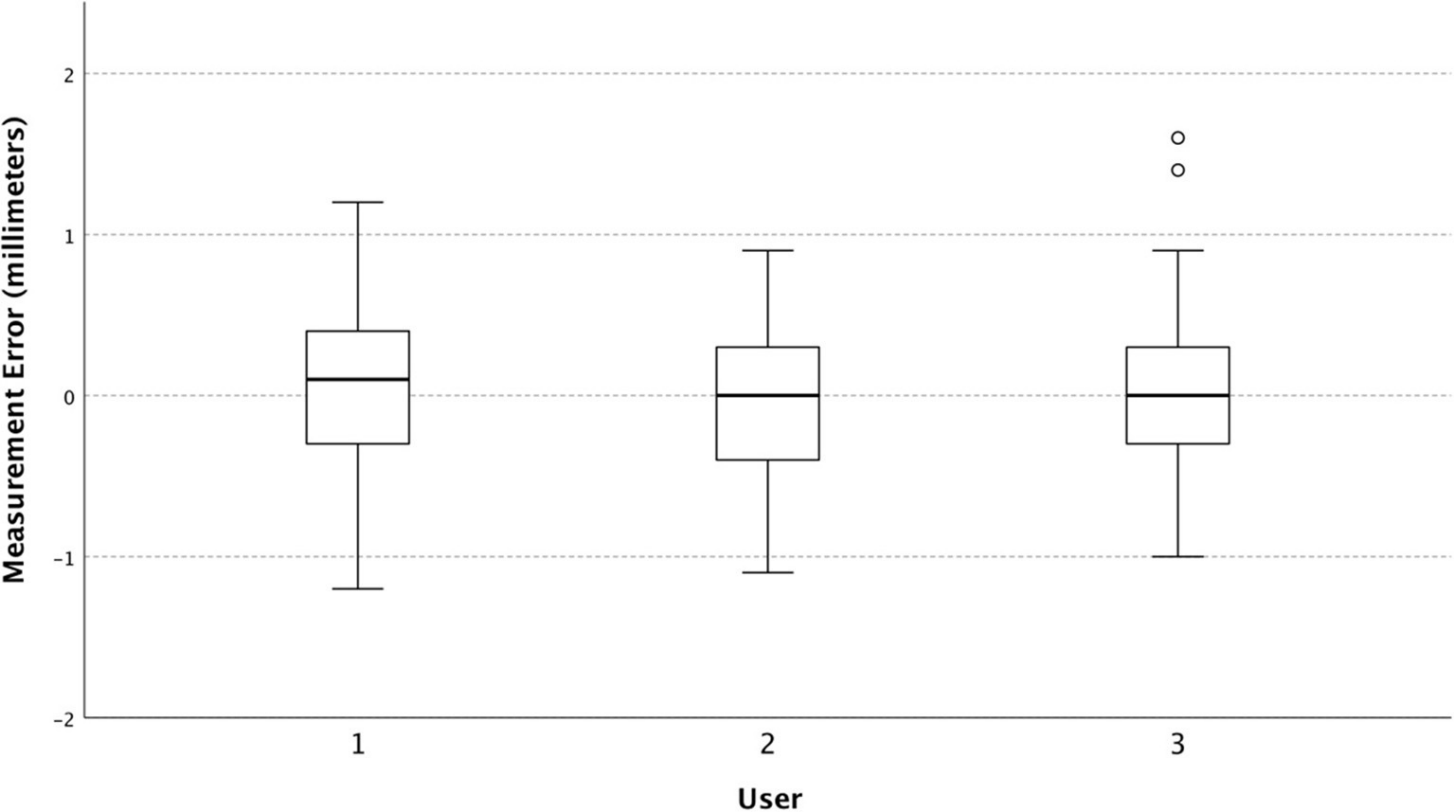
Box and whisker plot of measurement error (individual observed values minus predicted) stratified by the ultrasound device.

The mean squared error is presented as a composite measure of both the variance and bias (mean error) of the ultrasound devices, as both are important measures of device performance. We found that the Philips Lumify device outperforms the two Sonosite machines, with the IVIZ performing the worst. The devices are similar concerning their mean errors but are quite different in their variances (Figure 2). This suggests that different aspects of the ultrasound devices affect the users' precision but do not cause one device to over-or under-estimate the diameter. The IVIZ, which had the worst precision, only had a standard deviation of its error of 0.076 mm larger than the M-turbo machine. When determining a 3–8 mm structure's size (13, 19), a <0.1 mm increase in error would likely not make much of a clinically significant difference. For example, a 0.075 mm measurement error is much lower than previously reported values of interobserver variability of ±0.2 mm (30). Furthermore, this small measurement error is insignificant in comparison to the variation in reported ONSD measurement threshold values which vary as much as 1 mm in the literature (31). Some authors contend that a trend in increasing repeated ONSD measurements or extreme values is more useful in diagnosis to account for these measurement uncertainties (32). Based on the results of our current study, using one ultrasound unit for repeated measurement may be prudent so as to diminish between-device measurement variability.

**Figure 3:**
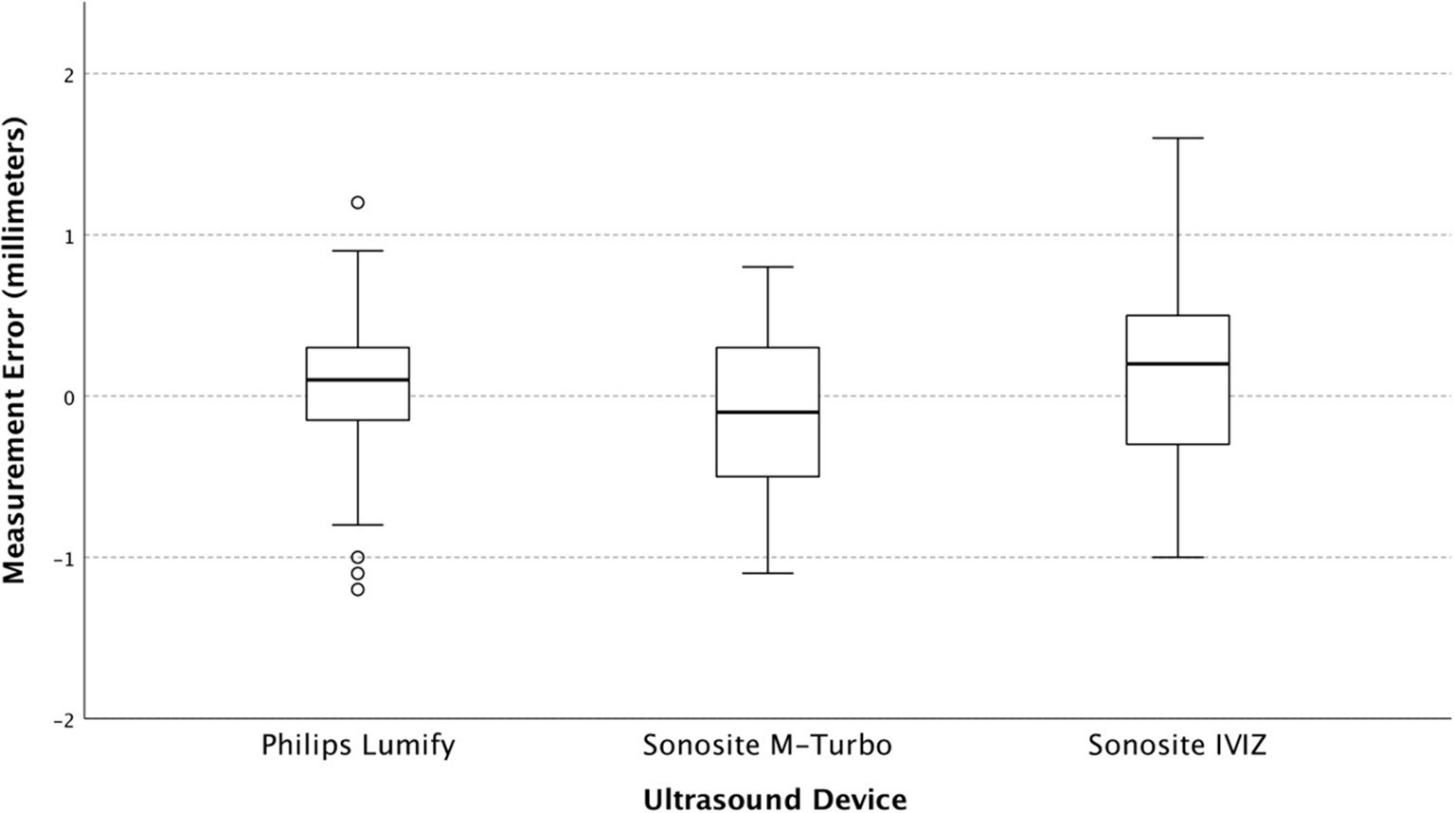
The Box and whisker plot of measurement error (individual observed values minus predicted) stratified by user.

The variance found in our study is smaller than what we have previously found in simulation models using conventional ultrasound (18). This is likely due to our exclusive reliance on expert sonographers as opposed to using novices. On analysis of our users, we found that they did not significantly differ with regards to their mean error or their variance when compared across all machines and model sizes (Figure 3), indicating that they all had a similar technique, and did not affect the differences we see between machines.

**Figure 4:**
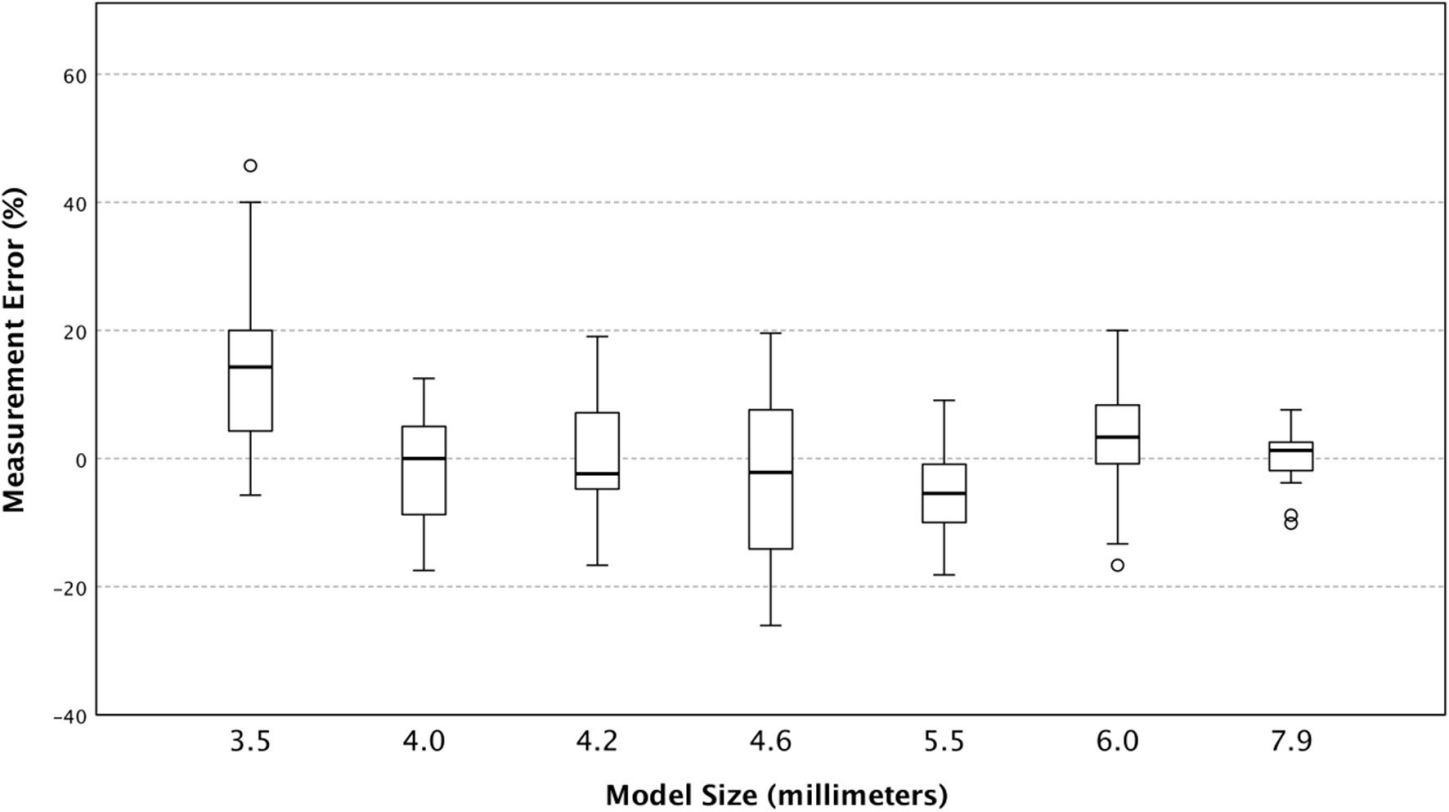
The Box and whisker plot of percent measurement error (individual observed values minus predicted) stratified by predicted size of model optic nerve sheath diameter.

Interestingly, when stratifying by ONSD model size, we find that the smallest model (3.5 mm) caused users to overestimate its size across all machines without a substantial corresponding increase in variance (Figure 4). This may be due to smaller structures inherently being more difficult to measure accurately and had we added another even smaller tube diameter to our study, we may have found this trend to continue.

One potential source of error in our model is that the materials used to simulate the ONS were slightly different between models. It is reasonable to assume that they would not all perform equally well when measured by ultrasound as they are likely to refract sonographic waves differently causing backscatter and reduced image clarity. We used tubing of various diameters from medical supplies made out of a variety of different plastics. However, when we analyzed and validated these models previously, we found that no model appeared to stand out as a poor performer (23). Again, in our current study, none of the models appeared to stand out as being a particular outlier, other than the smallest tube, which was composed of the same materials as many of the larger tubes. If the tubing composition did affect experts' ability to measure tube diameter, it was likely so small of an effect as to be undetectable in our current study.

Our model with known measurable tube sizes also highlights some of the difficulty of ultrasound determination of ONSD as a diagnostic test in general. In our study, experts in a controlled setting with no distractions and low variability in their repeated measurements over time, occasionally (rarely) over or under-estimated the ONS size by as much as 1.6 mm. For measurement of a structure with a cut-off value of 5–6 mm between normal and abnormal (13), a 1 mm difference can be a large clinically important discrepancy. It is important to remember that ONSD assessment is only one component of the clinical assessment of a patient with suspected IH, and that results must be interpreted in a greater clinical context. All diagnostic tests have false positives and negatives, and other *in vivo* studies have shown reasonable sensitivity and specificity of sonographic ONSD for IH as compared to other diagnostic modalities (13, 24, 33–35), indicating that in practice, measurement error this large is likely rare enough to not preclude ocular ultrasound for this purpose.

Comparison of three ultrasound devices produced some results that we were not expecting to measure. The Sonosite IVIZ overheated and crashed multiple times throughout the 3-h session, so its protective case was removed, and a portable fan was directed at it in order for it to function. This was not an issue for the Lumify or the M-turbo. The IVIZ battery life was also much shorter than that of the Lumify and could not be charged and used simultaneously, so two separate battery packs were used to complete the 3-h session. Most ultrasound machines would not be expected to function for such long continuous use in normal clinical applications, so this is likely not significant for *in vivo* applications.

Despite the importance of our findings, there are limitations to our study. Data comparing the ultrasound devices were measured on a simulation model in a controlled experimental setting. This was chosen by design to focus on the ultrasound devices themselves rather than external factors such as stressors in a clinical environment, patient variability, and time constraints. It also allows for a “gold standard” comparison against the measurable tube diameters used to simulate the ONS. However, it may be difficult to extrapolate the findings *in vivo*. We found low variance and measurement error, which may differ once clinical and patient factors are introduced. Our model itself may have also presented some errors of its own. The tubes may be more easily measured than the ONS of a real patient due to the lack of necessary concern for patient comfort, perpendicular tube position, and lack of artifact from ocular structures. Our model can only simulate the B-scan method of sonographic ONSD assessment, which is the most common method described in the literature, but is potentially inferior to a more recent method described (13, 36). Benefits of this method include ease of application, as the newer method requires careful image acquisition accounting for multiple structures in the orbit. Alternate methods such as trends in measurement over time (32), as well as automated measurement techniques have also been investigated with promising results (37–39).

Another limitation to our study is inherent to the popular B-scan ultrasound technique used to acquire images. Due to non-standardized gain settings used to acquire images, a “blooming effect” occurs, where larger gains cause the ONS to appear larger, and smaller gains cause it to appear smaller (40). This effect leads to artifacts (41) and decreased repeatability of measurements between ONSD studies (42). As in real life, gain was not standardized across devices during the present study, which may account for some of the variation observed. An alternate measurement technique, called the A-scan technique, uses a non-focused probe, applied to the open, anesthetized eye, and is recommended as a superior alternative to the B-scan technique, as it is free from this blooming effect (43). Whether the present simulation model and the pocket ultrasound devices can accurately apply this A-scan technique requires further research.

Images obtained using the Lumify attached to the Samsung smart tablet also subjectively had more clarity than the other two devices. This is partially explained by the tablet's high resolution, which has an 8-inch screen with 1,536 × 2,048 pixels (44). The iViz has a 7-inch screen with 1,920 × 1,200 pixels (45), and the M-Turbo has a 10.4-inch screen with 800 × 600 pixels (46). It is no surprise that the image was sharper on the Samsung tablet using the Lumify device compared to the M-turbo, however, the iViz screen resolution is similar to the tablet, so the increased image clarity of the Lumify may be related to other factors such as software interface, probe performance, and other hardware. These all may have been contributing factors that affected the variance in measurements observed between the ultrasound devices and lead to some of the improved performance of the Lumify over the others. It is unclear if these results would change if using the Lumify device on another smart tablet, however, it has been previously suggested that screen resolution and pixelization have minimal effects on measurement accuracy for sonographic measurements (47). Finally, we used a relatively small group of experts. As ONSD measurement is still considered to be experimental due to debate around appropriate cutoff values between normal and abnormal (48), only a limited number of practitioners in our center have sufficient experience with this technique.

Most pocket ultrasounds are not currently approved in the United States or Canada for ONSD measurement in humans. The existing devices generally do not have the required manufacturer presets to make these measurements safely. The recommended settings for ONSD ultrasound in human subjects require a TI ≤ 1 and MI ≤ 0.23 (49). The MI and TI are based upon the frequency output of the device, the depth of exam, and the tissue properties being examined. Manufacturer presets use software to limit the ultrasound focal length and energy output so that they do not exceed these safety thresholds (41). During our data collection, the Lumify and IVIZ did not have ocular ultrasound manufacturer presets, so we did not maintain their MI below the safety threshold during our study, which was part of the rationale to use a simulation model rather than human subjects for our measurements. These devices do however have the capability to display the MI and TI which could potentially be adjusted to maintain their levels below the recommended safety thresholds, so they could potentially be used in the future for this purpose. In fact, subsequent to our data collection, Sonosite IVIZ's newest firmware does provide for an ophthalmic preset using certain probes. The Philips device does not yet have ophthalmic presets (45, 50). We caution users that ophthalmic presets must be developed before pocket ultrasound units can be safely used in human subjects for ocular ultrasound.

## Conclusion

In summary, we found that the ultrasound devices differ in their bias and variance with the Lumify device performing the best and the IVIZ performing the worst, however not differing enough to be clinically relevant. We conclude that these pocket ultrasounds do appear to have the resolution to make an accurate determination of ONSD as well as the full-sized portable unit. However, each device is different and new models must be tested prior to use for this purpose. While it is unclear whether ultrasound will ever replace intracranial monitors in the diagnosis and management of IH, pocket ultrasound devices are a promising tool for the assessment of neurocritically ill patients, especially when access to the gold standard is limited, such as in rural or remote settings. As pocket ultrasound continues to expand beyond the tertiary care centers and enters the non-university hospital world, this has the potential to become a real triage tool like FAST, eFAST, and other POCUS applications. Adequate safety settings and regulatory approval are however necessary first before these devices can be used in this matter for ONSD measurement.

## Meetings

This work was presented as a poster at the American College of Surgeons Clinical Congress in October 2020.

## Data Availability Statement

The raw data supporting the conclusions of this article will be made available by the authors, without undue reservation.

## Ethics Statement

Ethical review and approval was not required for the study on human participants in accordance with the local legislation and institutional requirements. The participants provided their written informed consent to participate in this study.

## Author Contributions

LG, GJ, TJ, and AD were involved in data collection. All authors were involved in study design, data analysis, manuscript composition, read, and approved the final manuscript. All authors contributed to the article and approved the submitted version.

## Funding

FAZ receives research support from the Manitoba Public Insurance (MPI) Neuroscience Research Endowment/Operating Fund, the United States National Institutes of Health (NIH) through the National Institute of Neurological Disorders and Stroke (NINDS), the Canadian Institutes of Health Research (CIHR), the Canada Foundation for Innovation (CFI), the University of Manitoba VPRI Research Investment Fund (RIF), the University of Manitoba Rudy Falk Clinician-Scientist Professorship, the University of Manitoba Center on Aging Fellowship, the Winnipeg Rh Institute/University of Manitoba - Falconer Emerging Research Rh Award, and the Health Sciences Centre Foundation Winnipeg.

## Conflict of Interest

The authors declare that the research was conducted in the absence of any commercial or financial relationships that could be construed as a potential conflict of interest.

## Publisher's Note

All claims expressed in this article are solely those of the authors and do not necessarily represent those of their affiliated organizations, or those of the publisher, the editors and the reviewers. Any product that may be evaluated in this article, or claim that may be made by its manufacturer, is not guaranteed or endorsed by the publisher.
